# Supplementary material for: Ruminococcaceae_UCG-013 Promotes Obesity Resistance in Mice
Source: Biomedicines. 2022 Dec 16;10(12):3272. doi: 10.3390/biomedicines10123272 (PMC9776008; doi:10.3390/biomedicines10123272)
Supplement: Supplementary file 1 [file biomedicines-10-03272-s001.zip › biomedicines-2030434-supplementary.pdf]

Supplementary Materials

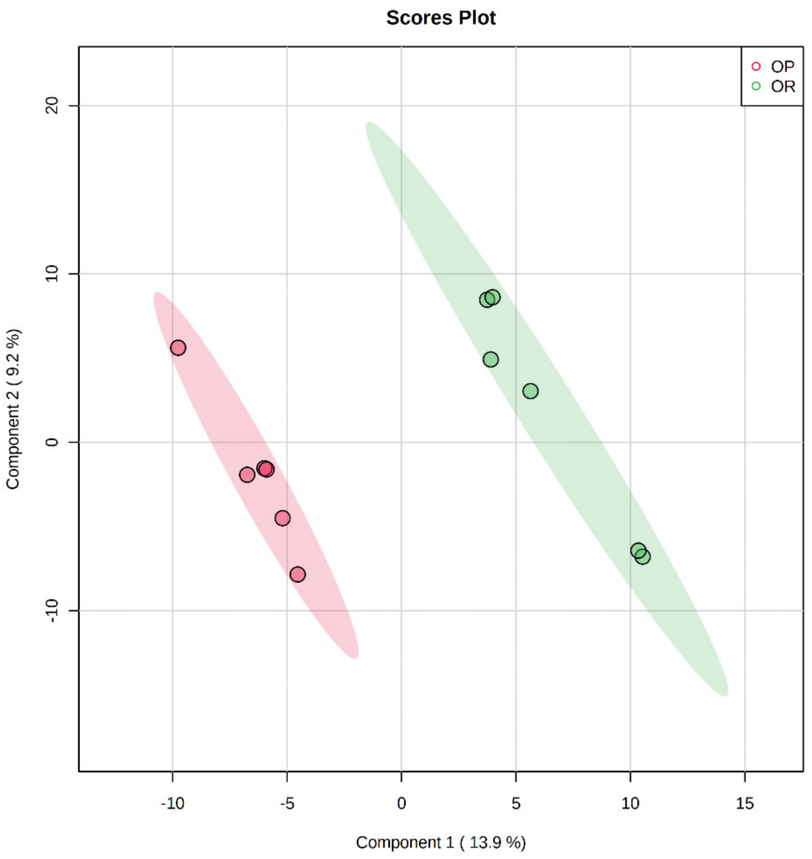

Figure S1. Partial least-square discriminant analysis (PLS-DA) scores plot of OTU level showing the groupings of OP (red) group and OR (green).

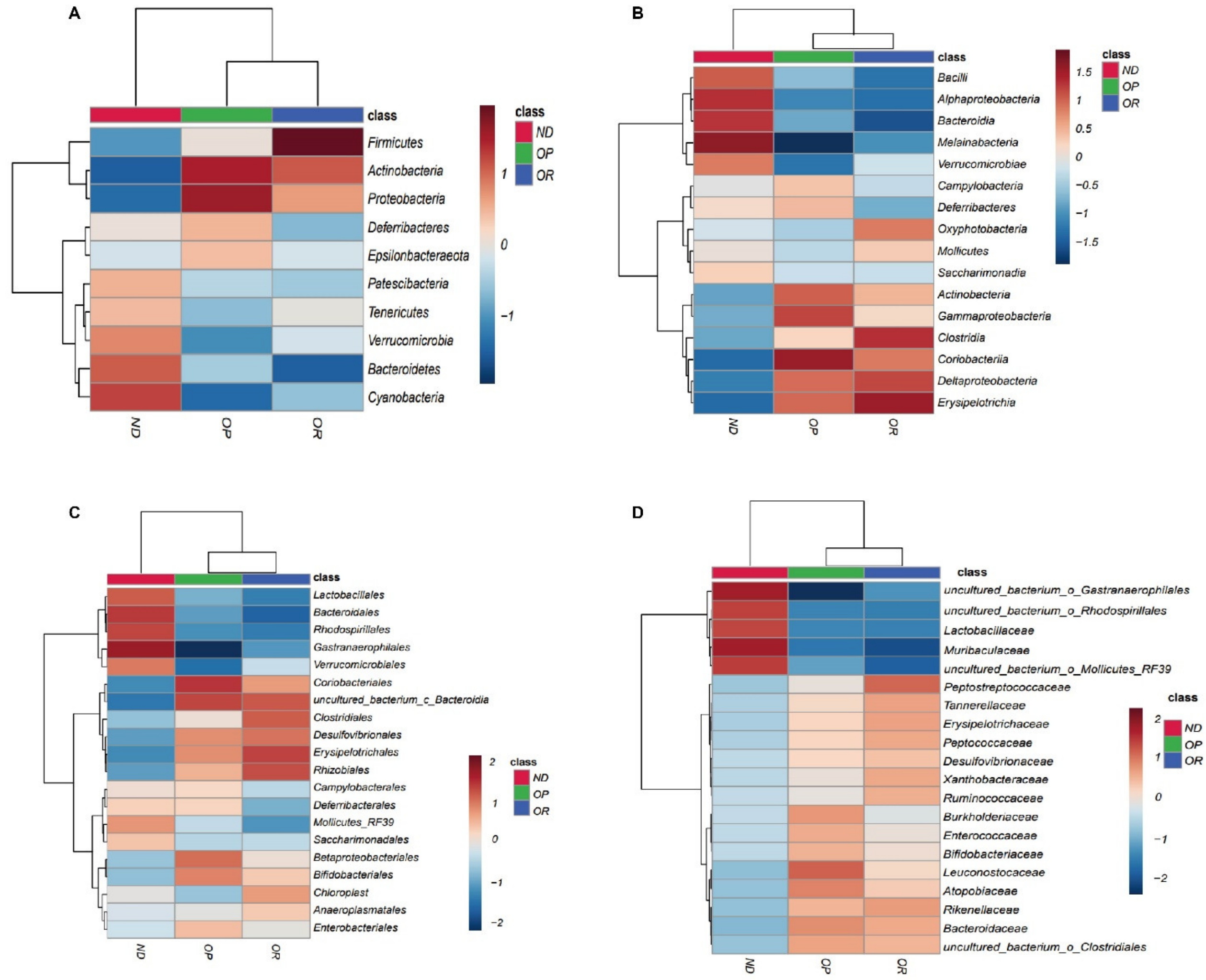

Figure S2. Comparison of the relative abundance of bacterial groups in the OP versus OR groups. (A) Heatmap of the Phylum level among different groups. (B) Heatmap of the Class level among different groups. (C) Heatmap of the Order level among different groups (only the top 20 taxa with the largest mean relative abundance are shown) (D) Heatmap of the Family level among different groups (only the top 20 taxa with the largest mean relative abundance are shown).

Table S1. Ingredients list for high fat feeds.

Product	High-Fat Diet(D12492)	
	gm%	kcal%
Protein	26.2	20
Carbohydrate	26.3	20
Fat	34.9	60
Total (kcal/gm)	5.24	
ingredients	gm	kcal
Casein,80 Mesh	200	800
L-Cystine	3	12
Corn Starch	0	0
Maltodextrin 10	125	500
Sucrose	68.8	275.2
Cellulose,BW 200	50	0
Soybean Oil	25	225
Lard*	245	2205
Mineral Mix,S10026	10	0
Dicalcium Phosphate	13	0
Calcium carbonate	5.5	0
Potassium Citrate, H_2O	16.5	0
Vitamin Mix,V10001	10	40
Choline Bitartrate	2	0
FD& C Blue Dye#1	0.05	0
Total	773.85	4057

*Typical analysis of cholesterol in lard=0.95mg/gram
Cholesterol(mg)/4057kcal=232.8
Cholesterol(mg)/kg=300.8

Table S2 Primer sequences

Target Bacterial	Sequence (5’-3’)	Sequence Length
Ruminococcaceae_UCG-013	F：TGGAGCAAACCCCCAAAAA<br>R：TACAAGGCCCGGGAACGTA	122
